# Dentofacial Characteristics in Patients With Epidermolysis Bullosa—A Cross‐Sectional Study

**DOI:** 10.1002/cre2.70478

**Published:** 2026-10-06

**Authors:** Sebastián Véliz, Susanne Krämer, Colomba Besa‐Witto, Pedro Diz‐Dios

**Affiliations:** ^1^ Department of Periodontology, Dental Clinic Sigmund Freud University Vienna Austria; ^2^ Special Care Dentistry Clinic, Faculty of Dentistry University of Chile Santiago Chile; ^3^ Medical‐Surgical Dentistry Research Group, Health Research Institute of Santiago de Compostela, School of Medicine and Dentistry Santiago de Compostela University Santiago de Compostela Spain; ^4^ Department of Dermatology, Medical Faculty and Medical Centre University of Freiburg Freiburg Germany

**Keywords:** cross‐sectional studies, epidermolysis bullosa, malocclusion, rare diseases

## Abstract

**Objectives:**

Epidermolysis bullosa (EB) is a rare genetic disease characterized by skin and mucosal fragility caused by pathogenic variants in structural protein genes. Malocclusions have been previously described in patients with EB, mainly in case reports, with no cohort studies characterizing dentofacial characteristics across all EB types. This study aims to describe and compare the dentofacial characteristics of patients with EB across all types in a nationally representative cohort.

**Material and Methods:**

A cross‐sectional study examined 101 patients with genetically confirmed EB diagnosis at a national reference center (2023 and 2024). Occlusal and extraoral characteristics were assessed by calibrated examiners (Cohen's Kappa: Intrarater 0.925, Interrater 0.931), including overjet, Angle's molar class, overbite, midline deviation, posterior transverse relationship, dental anomalies, crowding, facial profile, type, and symmetry. Associations were examined using Fisher's exact test with Cramér's *V* as effect size measure, and Kruskal–Wallis test with epsilon‐squared; *p* values were adjusted using the Benjamini–Hochberg false discovery rate procedure.

**Results:**

The sample included: EB simplex (EBS, *n* = 26), junctional EB (JEB, *n* = 6), dominant dystrophic EB (*n* = 18), recessive dystrophic EB (RDEB, *n* = 49), and kindler EB (*n* = 2). RDEB presented the highest prevalence of posterior transverse anomalies (74.5%, 95% CI 60.5–84.7; Cramér's *V* = 0.504, *p*[FDR] < 0.001) and altered overbite (Kruskal–Wallis *p* = 0.022, *ε* ^2^ = 0.106), with a significant difference compared to EBS (post‐hoc adjusted *p* = 0.022). All participants with JEB presented dental anomalies (100%, 95% CI 61.0–100.0; Cramér's *V* = 0.762, *p*[FDR] < 0.001). 53.1% of RDEB participants presented three or more dentofacial features coherent with growth restriction.

**Conclusions:**

Dentofacial anomalies vary according to the EB type. RDEB showed the highest prevalence of posterior transverse anomalies and altered overbite, and JEB presented universal dental anomalies. These findings support prioritizing and tailoring orthodontic screening by EB type. Future studies should address genotype–phenotype correlations, multicentre designs, and the relationship between dentofacial anomalies and orofacial functional impairments.

## 1 Introduction

Epidermolysis bullosa (EB) is a group of rare genetic diseases characterized by skin and mucosal fragility, resulting in ulcers and bullae due to abnormal proteins functioning at the dermo‐epidermal junction (Has et al. 2020). These genodermatoses present a highly variable phenotype associated with variants in at least 16 structural genes (Has et al. 2020; Bardhan et al. 2020). The current classification scheme considers four types: EB simplex (EBS), junctional EB (JEB), dystrophic EB (DEB), and kindler EB (KEB). These four main types are subdivided into more than 30 subtypes. Some of them share a common genetic origin but present extremely different phenotypes, such as dominant DEB (DDEB) and recessive DEB (RDEB), both caused by genetic variants in *COL7A1*. Additionally, since the 2020 EB classification, a new term was introduced, including “other skin conditions with skin fragility.” This new term includes five conditions, for example, peeling skin disorders (PSDs), which were previously diagnosed as EB variants but currently are not considered EB (Has et al. 2020). The overall prevalence of EB varies according to the country, with reports ranging from 5.5 per million inhabitants in Romania (Dănescu et al. 2015), 9.6 in Chile (Palisson et al. 2024), 11.07 in the United States (Fine 2016) to 54 in Germany (Has et al. 2023).

Even though EB is considered a skin disease, its clinical features affect several structures, including the orofacial region. The clinical presentation varies according to the EB type/subtype and its severity. The main oral features of all types are oral mucosal wounds and bullae (Krämer et al. 2020). Patients with EBS and DDEB present history of oral ulcers and blisters, with a generally milder orofacial phenotype compared to other EB types (Krämer et al. 2020). Patients with JEB present with perioral granulation tissue, amelogenesis imperfecta (hypoplastic generalized or pitted), teeth retention, crown resorption, and gingival overgrowth (Krämer et al. 2020; Wright et al. 1993). Patients with RDEB present oral strictures causing ankyloglossia, vestibule obliteration and microstomia, loss of tongue papillae and palatal rugae, and high caries prevalence influenced by poor oral hygiene due to factors such as pseudosyndactyly as well as pain and mouth opening limitations (Krämer et al. 2020). Meanwhile, KEB is characterized by early onset periodontal diseases, oral strictures, and pitting amelogenesis imperfecta (Krämer, Hillebrecht, Wang, et al. 2020, Krämer et al. 2024b).

Orthodontic manifestations and treatment in patients living with EB have been primarily reported in case reports and case series limited to a specific subtype, such as RDEB (Goldschmied 1999; Pacheco and Marques de Sousa Araugio 2008; Portillo Nava et al. 2014; Blanchet et al. 2021; Véliz et al. 2020, 2024; Véliz Méndez, Baeza, et al. 2022a, Véliz Méndez et al. 2023b). Reported malocclusions comprise a variety of clinical presentations, such as severe crowding, maxillary hypoplasia, and posterior and anterior crossbite (Krämer et al. 2020; Goldschmied 1999; Pacheco and Marques de Sousa Araugio 2008; Portillo Nava et al. 2014; Blanchet et al. 2021; Véliz et al. 2020, 2024; Véliz Méndez, Baeza, et al. 2022a, Véliz Méndez et al. 2023b; Shah et al. 2002). Regardless of the increasing access to dental treatment in patients with EB (Krämer, Hillebrecht, Bekes, et al. 2024a), orthodontic features across all EB types have not been systematically characterized in a cohort study, with no study encompassing the full spectrum of EB types in a single cohort. This study aims to describe the dentofacial characteristics of patients living with EB, using a nationally representative cohort.

## 2 Methods

A cross‐sectional study was conducted at the Faculty of Dentistry of the University of Chile, the national reference center for dental care of patients with EB, in association with the “Dystrophic Epidermolysis Bullosa Research Association” (DEBRA) Chile, between January 2023 and December 2024. A comprehensive clinical assessment, including a specially designed orthodontic characteristics appendix, was conducted during the patient's regular oral health appointment. Given the low prevalence of EB, a census‐based approach was adopted, aiming to include all patients with EB diagnosis confirmed by genetic test attending the dental clinic during this time.

No funding was received for this project. Ethical approval was obtained at the Ethics Committee of the Ethics Committee of the North Metropolitan Health Service, Chile (002/2023). All participants were asked to sign a consent to participate. In the case of minors, both, the patient and their guardian, were asked to sign a consent to participate. Participants also consented to include their photos and information in this article. This project followed the Strengthening the Reporting of Observational Studies in Epidemiology (STROBE) guidelines for cross‐sectional studies (von Elm et al. 2007).

Inclusion criteria considered a genetically confirmed diagnosis of EB, agreeing to participate in this project, no age limit, and the capability of cooperating and following instructions. No additional criteria were applied to include or exclude participants.

The examination included the intermaxillary sagittal (overjet and Angle's molar relation), transversal (midlines and posterior relation including crossbite and scissors bite) and vertical (open bite/overbite) relationships, intramaxillary anomalies such as crowding and dental anomalies (agenesis, retention, supernumerary, shape and size anomalies, and transposition). Extraoral clinical assessment was also performed, including facial profile (straight, concave, or convex profile), facial type (normodivergent, hypodivergent, or hyperdivergent), and transverse symmetry (symmetrical, asymmetrical with mandible shifted left or right). Due to the complexity of oral conditions, particularly in RDEB, such as mucosal fragility, vestibule obliteration and microstomia (most severely affected participant with only 6 mm of maximal mouth opening), the use of dental impressions or intraoral scanners was not considered routinely feasible across the cohort; therefore, crowding was assessed only as present/absent through direct intraoral visualization (third molars were not considered).

To reduce the risk of bias, several considerations were implemented. The invitation to participate was made through DEBRA Chile, and all registered patients attending dental check‐ups were eligible to participate. Data were collected for nearly all patients, as declared in each Table (Valid/Missing). The intraoral examination was performed by an orthodontist (S.V.) and a special care dentist (C.B.) previously calibrated. Agreement for each variable was calculated using Cohen's Kappa (unweighted; intrarater: 0.925; interrater: 0.931); in case of disagreement, a third special care dentist (S.K.) was consulted to reach consensus.

### 2.1 Statistical Analysis

As this is a descriptive cross‐sectional study, statistical analyses were used to characterize the distribution of variables and identify differences across EB types and subtypes, not to establish causal relationships. Categorical variables were summarized as counts and percentages with 95% Wilson confidence intervals (CIs), which perform well with small samples and proportions near 0% or 100%. Continuous variables (overjet and overbite) did not meet normality assumptions and were summarized as medians with interquartile ranges. Associations between EB types (EBS, JEB, DDEB, and RDEB; KEB was excluded due to its sample size of two and was descriptively reported) and categorical variables were assessed with Fisher's exact test, and the strength of association quantified with Cramér's V (0.1, 0.3, and 0.5 indicating small, medium, and large effects, respectively). To account for multiple comparisons across categorical outcomes, *p* values were adjusted using the Benjamini–Hochberg false discovery rate procedure, with both unadjusted and adjusted *p* values reported. For continuous variables, Kruskal–Wallis test was used to evaluate differences across the same EB types, using epsilon‐squared (*ε* ^2^) as the effect size measure. In case the global test was significant, a pairwise post‐hoc comparison with Bonferroni adjustment was performed. Outcomes were analyzed at participant level, except molar relationship, that was recorded by hemiarch side, but summarized per participant to avoid treating both sides as independent observations. Missing data were reported per variable. A two‐sided *p* value < 0.05 was considered statistically significant. All analyses were conducted using RStudio (version 2026.01.01 + 403).

## 3 Results

### 3.1 Sample Description

A total of 101 participants were examined, including 26 EBS (localized [*n* = 3], intermediate [*n* = 18] and intermediate with muscular dystrophy [*n* = 5]), 6 JEB (severe [*n* = 3] and intermediate [*n* = 3]), 18 DDEB (localized [*n* = 14], and pruriginosa [*n* = 4]), 49 RDEB (severe [*n* = 25], intermediate [*n* = 21], inversa [*n* = 2], and localized [*n* = 1]), and 2 KEB. Gender distribution of the complete sample included 54 male (53.5%) and 47 female (46.5%), with no participant identifying as diverse, and a mean age of 22.46 ± 15.3 (ranging from 2 to 74 years). A total of six patients were excluded: refusal to participate (*n* = 3), lack of genetic confirmation of EB (*n* = 1), and inability to follow instructions during examination (*n* = 2) (Supporting Information S1: Figure S1). Out of a universe of 192 eligible individuals, 101 participants were included. Assuming a 95% confidence level and an expected proportion of 50%, this sample size provides an estimated margin of error of approximately 6.7%.

### 3.2 Intermaxillary Relationship Assessment

#### 3.2.1 Sagittal Plane

Overjet was assessed in 93 participants, with an overall median of 3.0 (1.0–4.0) mm, and 67.7% (*n* = 63) of them presenting values between 1 and 4 mm. The highest overjet values were observed in JEB (median 3.5 [3.0–4.0] mm) and DDEB (median 3.5 [2.0–4.0] mm), while the lowest values were observed in RDEB (median 2.0 [1.0–4.0] mm). The prevalence of affected overjet did not differ significantly across EB types (Fisher's exact test *p* = 0.712, *p*[FDR] = 0.712, Cramér's *V* = 0.133), with prevalences of 24.0% (95% CI 11.5–43.4) in EBS, 16.7% (3.0–56.4) in JEB, 38.9% (20.3–61.4) in DDEB, and 27.9% (16.7–42.7) in RDEB. Additionally, RDEB presented the highest percentage of anterior crossbite (*n* = 9; 20.9%). For eight patients, it was not possible to assess the overjet due to missing anterior teeth (Tables 1 and 2, Supporting Information S1: Tables S1– S3).

**Table 1:** Prevalence of dentofacial anomalies by EB type.

Plane	Variable (Presence of malocclusion)	EBS (*n/N*, %, 95% CI)	JEB (*n/N*, %, 95% CI)	DDEB (*n/N*, %, 95% CI]	RDEB (*n/N*, %, 95% CI)	Cramer *V*	*p* (Fisher)	*p* (FDR)
Sagittal	Overjet	6/25 − 24.0% (11.5–43.4)	1/6 − 16.7% (3.0–56.4)	7/18 − 38.9% (20.3–61.4)	12/43 − 27.9% (16.7–42.7)	0.133	0.712	0.712
Sagittal	Molar relationship	16/22 − 72.7% (51.8–86.8)	5/6 − 83.3% (43.6–97.0)	11/17 − 64.7% (41.3–82.7)	17/21 − 81.0% (60.0–92.3)	0.155	0.699	0.712
Transverse	Midline	7/24 − 29.2% (14.9–49.2)	2/6 − 33.3% (9.7–70.0)	8/18 − 44.4% (24.6–66.3)	23/44 − 52.3% (37.9–66.2)	0.199	0.301	0.501
Transverse	Posterior transverse	5/26 − 19.2% (8.5–37.9)	1/6 − 16.7% (3.0–56.4)	7/18 − 38.9% (20.3–61.4)	35/47 − 74.5% (60.5–84.7)	0.504	< 0.001	< 0.001
Vertical	Overbite	3/25 − 12.0% (4.2–30.0)	2/6 − 33.3% (9.7–70.0)	7/18 − 38.9% (20.3–61.4)	21/43 − 48.8% (34.6–63.2)	0.320	0.016	0.052
Intramaxillary	Crowding	14/26 − 53.8% (35.5–71.2)	2/6 − 33.3% (9.7–70.0)	8/18 − 44.4% (24.6–66.3)	32/49 − 65.3% (51.3–77.1)	0.201	0.273	0.501
Intramaxillary	Dental anomalies	2/26 − 7.7% (2.1–24.1)	6/6 − 100.0% (61.0–100.0)	1/18 − 5.6% (1.0–25.8)	1/48 − 2.1% (0.4–10.9)	0.762	< 0.001	< 0.001
Extraoral	Facial profile	15/26 − 57.7% (38.9–74.5)	2/6 − 33.3% (9.7–70.0)	8/18 − 44.4% (24.6–66.3)	34/49 − 69.4% (55.5–80.5)	0.234	0.142	0.356
Extraoral	Facial type	17/26 − 65.4% (46.2–80.6)	4/6 − 66.7% (30.0–90.3)	15/18 − 83.3% (60.8–94.2)	37/49 − 75.5% (61.9–85.4)	0.143	0.570	0.712
Extraoral	Facial symmetry	3/26 − 11.5% (4.0–29.0)	1/6 − 16.7% (3.0‐56.4)	1/18 − 5.6% (1.0–25.8)	10/49 − 20.4% (11.5–33.6)	0.163	0.466	0.666

*Note:* Cramer's *V*: 0.1 small, 0.3 medium, and 0.5 large. Reduced denominators (e.g., molar relationship in RDEB) reflect teeth absent due to prior extraction. KEB (*n* = 2) was excluded from inferential testing and reported descriptively: of the two participants only one was dentate ‐ normal overjet and overbite, bilateral Class III molar relationship, bilateral posterior crossbite, centered midlines, and pitting enamel abnormalities; extraoral features were normal and symmetrical. Cells: affected/assessed (*n/N*), % (95% Wilson CI). *p* (FDR): Benjamini–Hochberg‐adjusted across the categorical outcomes. Abbreviations: DDEB, dominant dystrophic EB; EB, epidermolysis bullosa; EBS, epidermolysis bullosa simplex; JEB, junctional EB; RDEB; recessive dystrophic EB.

**Table 2:** Overjet and overbite by EB type.

Variable (mm)	EBS median [IQR](*n*)	JEB median [IQR](*n*)	DDEB median [IQR](*n*)	RDEB median [IQR](*n*)	Kruskal–Wallis (*p*)	*ε* ^2^
Overjet	3.0 [2.0–4.0] (*n* = 25)	3.5 [3.0–4.0] (*n* = 6)	3.5 [2.0–4.0] (*n* = 18)	2.0 [1.0–4.0] (*n* = 43)	0.313	0.039
Overbite	2.0 [1.0–3.0] (*n* = 25)	2.0 [2.0–2.8] (*n* = 6)	3.0 [1.2–3.8] (*n* = 18)	3.0 [2.0–6.0] (*n* = 43)	0.022	0.106

*Note:* KEB excluded from the Kruskal–Wallis test. epsilon‐squared: 0.01 small, 0.06 medium, and 0.14 large. Post‐hoc pairwise comparisons with Bonferroni adjustment revealed a significant difference in overbite between RDEB and EBS (adjusted *p* = 0.022); all other comparisons were not significant. Values: median [IQR] (*n*).

Abbreviations: DDEB, dominant dystrophic EB; EB, epidermolysis bullosa; EBS, epidermolysis bullosa simplex; JEB, junctional EB; RDEB, recessive dystrophic EB.

Angle's molar relation (Angle 1899) was evaluated bilaterally in 94 participants (188 hemiarch assessments); for inferential testing, molar relationship was summarized at participant level. Of the assessable hemiarchs, 46 (24.4%) corresponded to Class III, 42 (22.3%) to Class I and 35 (18.6%) to Class II. A total of 65 assessments (34.6%) could not be performed due to previous extraction of the first molar, with this finding being predominant in RDEB participants. Additionally, seven young patients were excluded because their first permanent molar had not yet erupted (Table 1, Supporting Information S1: Tables S1 and S2).

#### 3.2.2 Transversal Plane

The midline coincidence was assessed in 93 participants, coinciding in 57% (*n* = 53). The most prevalent group of non‐coinciding midlines were RDEB (52.3%, 95% CI 37.9–66.2, *n* = 23) and DDEB (44.4%, 95% CI 24.6–66.3, *n* = 8), although differences across EB types were not statistically significant after FDR adjustment (Fisher's exact test *p* = 0.301, *p*[FDR] = 0.501, Cramér's *V* = 0.199). For eight patients, it was not possible to assess the midline relationship due to missing anterior teeth (Table 1, Supporting Information S1: Tables S1 and S4).

The posterior transverse relationship was evaluated in 98 patients, with half of the participants showing no posterior transverse malocclusion (*n* = 49, 50.0%). Significant differences were found across EB types (Fisher's exact test *p* < 0.001, *p*[FDR] < 0.001, Cramér's *V* = 0.504), indicating a large effect. RDEB presented the highest prevalence of posterior transverse anomalies (*n* = 35/47, 74.5%, 95% CI 60.5–84.7), compared to 38.9% (95% CI 20.3–61.4) in DDEB, 19.2% (95% CI 8.5–37.9) in EBS, and 16.7% (95% CI 3.0–56.4) in JEB. In three patients, the transversal relationship could not be assessed due to missing posterior teeth (Table 1, Supporting Information S1: Tables S1 and S4).

#### 3.2.3 Vertical Plane

Overbite was assessed in 93 participants, with 64.5% (*n* = 60) presenting values between 1 and 4 mm, with an overall median of 3.0 (2.0–4.0) mm. Overbite differed significantly across EB types (Kruskal–Wallis test *χ* ^2^[3] = 9.62, *p* = 0.022, *ε* ^2^ = 0.106), indicating a medium effect. RDEB participants presented the highest overbite (median 3.0 [2.0–6.0] mm), followed by DDEB (median 3.0 [1.2–3.8] mm), JEB (median 2.0 [2.0–2.8] mm), and EBS (median 2.0 [1.0–3.0] mm). Post‐hoc pairwise comparison with Bonferroni adjustment showed significant difference between RDEB and EBS (adjusted *p*‐value = 0.022), while all other comparisons were non‐significant. The prevalence of affected overbite also showed a trend across EB types (Fisher's exact test *p* = 0.016, *p*[FDR] = 0.052, Cramér's *V* = 0.320), though this did not reach significance after FDR adjustment. For eight patients, overbite assessment was not possible due to missing anterior teeth (Tables 1 and 2, Supporting Information S1: Tables S1, S5 and S6).

The posterior vertical relationship could not be systematically assessed, particularly in the RDEB group, due to the combined effect of severe microstomia, high prevalence of missing posterior teeth, and the absence of dental casts. This assessment was therefore excluded from the comparative analysis.

### 3.3 Intramaxillary Assessment

Dental crowding was assessed in all 101 participants, being present in 56 patients (55.4%). The highest prevalence was observed in RDEB (*n* = 32/49, 65.3%, 95% CI 51.3–77.1), while the lowest prevalence was observed in JEB (*n* = 2/6, 33.3%, 95% CI 9.7–70.0), with no significant differences across EB types (Fisher's exact test *p* = 0.273, *p*[FDR] = 0.501, Cramér's *V* = 0.201).

Dental anomalies were examined in 99 patients, with 89 (89.9%) showing no anomalies. Significant differences were found across EB types (Fisher's exact test, *p* < 0.001, *p*[FDR] < 0.001, Cramér's *V* = 0.762), indicating a very large effect. All JEB participants (*n* = 6/6, 100%, 95% CI 61.0–100.0) presented one or more dental anomaly, including shape anomalies (enamel hypoplasia due to syndromic amelogenesis imperfecta) and eruption complications (retentions and crown resorption, also associated with syndromic amelogenesis imperfecta), compared to 7.7% (95% CI 2.1–24.1) in EBS, 5.6% (95% CI 1.0–25.8) in DDEB, and 2.1% (95% CI 0.4–10.9) in RDEB (Table 1, Supporting Information S1: Tables S1 and S5).

### 3.4 Extraoral Assessment

Extraoral characteristics were assessed for the complete sample (*n* = 101). Facial profile did not differ significantly across EB types (Fisher's exact test *p* = 0.142, *p*[FDR] = 0.356, Cramér's *V* = 0.234). A straight profile was observed in 40.6% of participants (*n* = 41), while the highest prevalence of concave profiles was observed in EBS (*n* = 12, 46.2%, 95% CI 29.9–64.9) and RDEB (*n* = 21, 42.9%, 95% CI 29.7–57.1), while convex profiles were most prevalent in RDEB (*n* = 13, 26.5%, 95% CI 16.3–40.1).

Facial type did not differ significantly across EB types (Fisher's exact test *p* = 0.570, *p*[FDR] = 0.712, Cramér's *V* = 0.143). The most prevalent facial type was hypodivergent (*n* = 45, 44.6%), with more than half of RDEB participants presenting this pattern (*n* = 27, 55.1%). On the contrary, 44.4% of DDEB participants (*n* = 8) presented a hyperdivergent profile.

Facial symmetry did not differ significantly across EB types (Fisher's exact test *p* = 0.466, *p*[FDR] = 0.666, Cramér's *V* = 0.163). Most patients showed a symmetrical frontal analysis (*n* = 86, 85.1%), with only 15 patients showing some type of asymmetry (14.9%, 95% CI 9.1–23.3). The highest prevalence of asymmetry by subtype was observed in EBS intermediate with muscular dystrophy, with 60% of affected participants (*n* = 3/5, 95% CI 23.1–88.2) (Table 1, Supporting Information S1: Tables S1 and S7).

An exploratory sensitivity analysis, restricted to participants with permanent dentition (*n* = 77; age ≥ 12 years), confirmed the robustness of the main findings (Supporting Information S1: Table S8).

## 4 Discussion

This study provides the most comprehensive characterization to date of dentofacial anomalies across all EB types in a single cohort, confirming the high burden of malocclusion in this population. Participants with RDEB, particularly those with the severe and intermediate subtypes, exhibited the highest prevalence of dentofacial anomalies, including posterior transverse anomalies (*Fisher's exact test*, *p* < 0.001, *p*[FDR] < 0.001, Cramér's *V* = 0.504) and increased overbite (*Kruskal–Wallis χ* ^2^[3] = 9.62, *p* = 0.022, *ε* ^2^ = 0.106), the latter with a significant difference especially between RDEB and EBS (adjusted *p* = 0.022). All patients with JEB presented dental anomalies, including shape anomalies and eruption complications (*Fisher's exact test*, *p* < 0.001, *p*[FDR] < 0.001, Cramér's *V* = 0.762), representing a very large effect size. Participants with EBS and DDEB showed comparatively milder anomalies across all variables. These findings show direct implications for orthodontic screening and referral priorities for those with RDEB and JEB, both types considered as “high risk of oral diseases,” along with KEB (Krämer, Hillebrecht, Bekes, et al. 2024a).

Most reports of malocclusions in EB are case reports (Goldschmied 1999; Pacheco and Marques de Sousa Araugio 2008; Portillo Nava et al. 2014; Blanchet et al. 2021; Véliz et al. 2020, 2024; Véliz Méndez, Baeza, et al. 2022a, Véliz Méndez et al. 2023b; Serrano et al. 2001), with just a few studies focusing on occlusal or skeletal characteristics, primarily in RDEB patients (Shah et al. 2002; De Benedittis et al. 2004; Korolenkova 2015). This cohort illustrates the diversity of malocclusion observed across different subtypes of EB (Figure 1).

**Figure 1:**
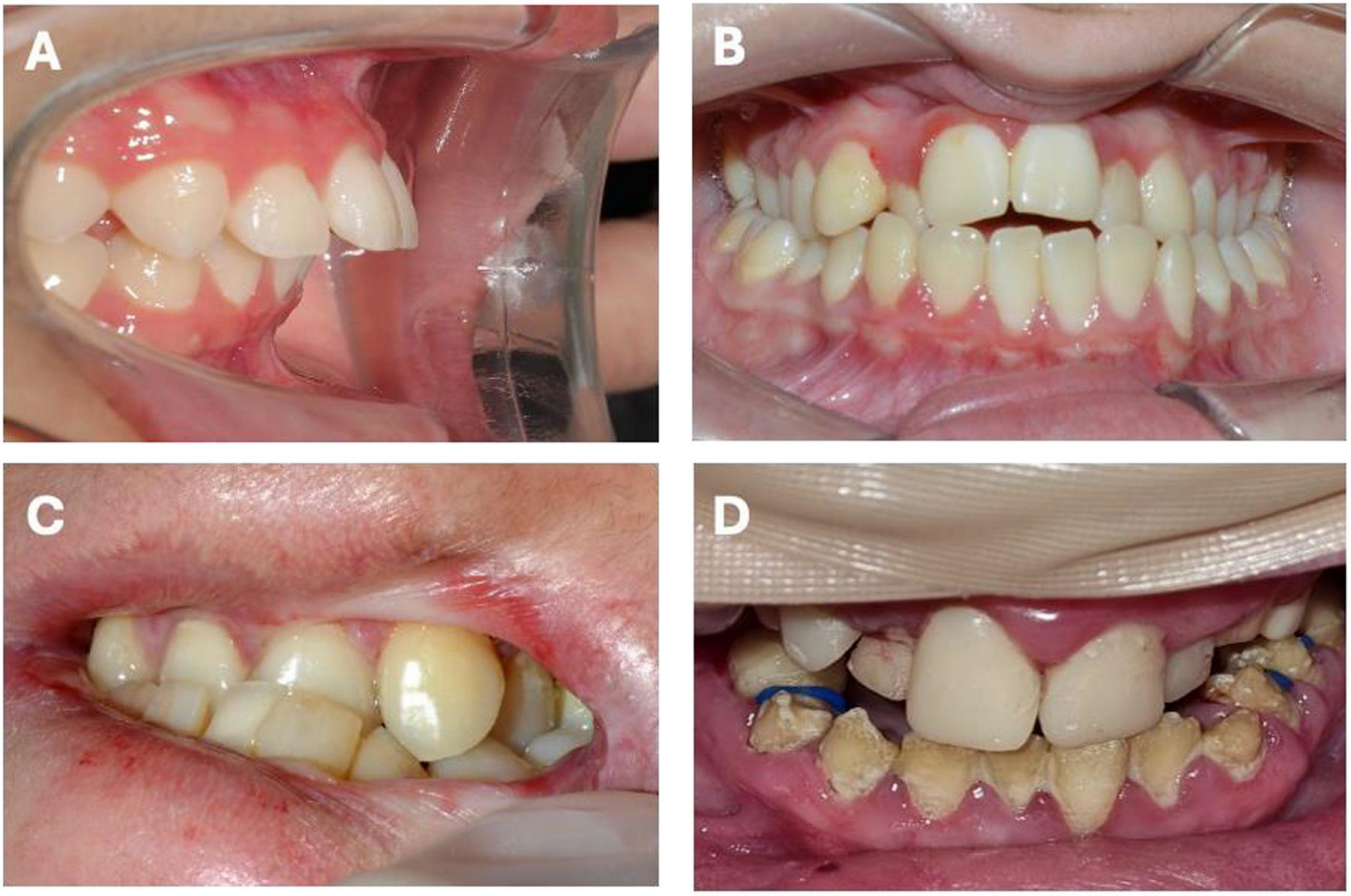
Malocclusions in patients with several types of EB. (A) Increased overjet in a patient with localized DDEB. (B) Anterior and posterior crossbite in a patient with localized DDEB. (C) Anterior and crossbite in a patient with severe RDEB. Additionally, the patient presents microstomia, vestibule obliteration, lip atrophy and lower incisors retroclination. (D) Anterior and posterior crossbite in a patient with intermediate JEB due to *LAMB3* variant. The patient presents Syndromic Amelogenesis Imperfecta (SAI).

The cohort included 101 patients, representing 52.6% of the EB patient population in the country, which estimated the total number of affected individuals to be 192 (Palisson et al. 2024). Compared to the general population, these findings show a higher prevalence of transverse malocclusions, particularly among RDEB patients (74.5%, 95% CI 60.5–84.7), in contrast to the findings of Pincheira et al. (21.7%), in the general Chilean population (Pincheira et al. 2018). While direct comparison is limited by differences in age range and study design, the magnitude of this difference, supported by a large effect size (Cramér's *V* = 0.504) suggests a substantially higher burden of transverse malocclusion in the EB population. These results are consistent with Shah et al. (2002), who reported a maxillary decreased size in patients with RDEB as a consequence of scars and malnutrition.

### 4.1 EB Simplex and Dominant Dystrophic EB

Orofacial characteristics and management of EBS and DDEB have long been overshadowed by more severe types of EB, such as JEB, RDEB, and KEB. Although severe EBS types do exist, the limited number of reports is mostly descriptive (Krämer et al. 2020). The most common feature shared by both types is the presence of oral ulcers and blisters, but they have a lower prevalence than in the more severe EB types (Krämer et al. 2020). Notably, a case of EBS with muscular dystrophy was reported to exhibit micrognathia and a high‐arched palate (Lee et al. 2017). In this cohort, absence of dentofacial anomalies was predominant in most analyzed variables, including overjet, overbite, midlines, and posterior transverse relationship, consistent with the non‐significant findings after FDR adjustment for these variables. Notably, three out of five participants with EBS intermediate with muscular dystrophy presented facial asymmetry (60.0%, 95% CI 23.1–88.2). This was the only EB subtype exhibiting a higher prevalence of asymmetry rather than symmetry. This trait may be attributed to the progressive muscular weakness expected in this EB subtype, which has been documented to start toward the end of the first decade, leading to progressive muscular atrophy (Chiavérini et al. 2010).

### 4.2 Junctional EB

There is scarce information regarding the occlusal and skeletal characteristics of JEB patients, primarily due to the high early mortality risk associated with this EB type, which is estimated to be 62% by the age of 15 years in severe JEB, making long‐term follow‐up particularly challenging (Fine et al. 2008). In this cohort, six patients with JEB due to *LAMB3* variants were assessed, with an average age of 12 years. This variant is associated with syndromic amelogenesis imperfecta, crown resorption, dental agenesis, retention/delayed tooth eruption, and taurodontism (Bardhan et al. 2020; Wright et al. 1993; Collins et al. 1999). All patients in this series (*n* = 6/6, 100%, 95% CI 61.0–100.0) presented at least one dental anomaly (Fisher's exact test, *p* < 0.001, *p*[FDR] < 0.001, Cramér's *V* = 0.762), including shape anomalies and eruption complications (crown resorption, retention, or impacted teeth). Additionally, one participant also presented a second lower premolar agenesis. Despite the small sample size, the very large effect size (Cramér's *V* = 0.762) supports the robustness of this finding. These findings align with previous evidence regarding dental anomalies, with a 100% prevalence of dental anomalies in JEB due to *LAMB3* variants (Wright et al. 1993). At the same time, previous reports highlight a high prevalence of external crown resorption in JEB patients due to *LAMB3* variants, with 90% of patients (*n* = 20) presenting at least one tooth with external crown resorption (Besa‐Witto et al. 2025), which is consistent with our results.

It is noteworthy that despite the previously described association between amelogenesis imperfecta and anterior open bite (Broutin et al. 2023) in the general population, in our JEB cohort only one patient presented a decreased overbite. Further studies in other EB cohorts should consider larger samples to evaluate different variants for JEB and, more thoroughly, assess the relationship between amelogenesis imperfecta and anterior open bite, including profile cephalometric radiographs and longitudinal assessments.

### 4.3 Recessive Dystrophic EB

Severe teeth crowding, posterior crossbite, and increased overjet have been previously described in case reports of patients with RDEB (Véliz et al. 2020; Serrano et al. 2001; De Benedittis et al. 2004). In 2002, Shah et al. (2002). evaluated 42 lateral cephalometric radiographs of RDEB patients aged between 4 and 18 years, assessing growth patterns and concluding that RDEB patients had smaller maxillary length, mandibular length, middle facial height, and lower facial height when compared to healthy controls. The authors proposed that feeding difficulties in RDEB patients affected overall growth, including craniofacial development, leading to severe dento‐alveolar disproportion. Other authors suggested that orofacial strictures and scarring might impact orofacial growth, including microstomia, peri‐oral granulation tissue, ankyloglossia, and vestibule obliteration (Krämer et al. 2020; Wright et al. 1991). Limitations in the orofacial function of RDEB patients have also been reported and proposed as contributing to orofacial abnormalities in RDEB (Stellingsma et al. 2011). These mechanisms are consistent with our findings. The high prevalence of posterior transverse anomalies in RDEB (74.5%, 95% CI 60.5–84.7; Cramér's *V* = 0.504) supports the restricted maxillary development in this subtype. Notably, the high rate of missing first molars in RDEB (34.6% of assessments not performable due to prior extraction) may also reflect the severity of dental disease in this group, and should be considered a clinically relevant finding beyond methodological limitation. The predominant hypodivergent facial pattern observed in this group (55.1%) may reflect the functional impact of strictures, such as microstomia and ankyloglossia, on the vertical facial development, restricting normal muscular activity and mandibular growth, as proposed by Shah et al. (2002). Additionally, overbite was significantly higher in RDEB compared to EBS (Kruskal–Wallis *χ* ^2^[3] = 9.62, *p* = 0.022, *ε* ^2^ = 0.106; post‐hoc adjusted *p* = 0.022), with RDEB presenting a median overbite of 3.0 (2.0–6.0) mm. Taken together, the co‐occurrence of posterior transverse anomalies, increased overbite, concave profile and hypodivergent facial type in RDEB suggests a phenotype consistent with restricted maxillary growth. To further explore this phenotype, co‐occurrence of these four features was assessed in RDEB participants, with 10/49 (20.4%) presenting all four simultaneously, 26 (53.1%) presenting three or more, and only one participant (2%) not presenting any of these features (RDEB intermediate), supporting the existence of a clinically coherent dentofacial phenotype in this subtype (Supporting Information S1: Figure S2). Further studies should explore the relationship between these anatomical findings and orofacial functional impairments due to strictures.

### 4.4 Kindler EB

KEB is the rarest type of EB, with approximately 250 cases reported since its first description (Has et al. 2020), making it particularly challenging to characterize this group. Krämer et al. described the orofacial features in a cohort of 36 KEB patients, with periodontal diseases being the most common trait, found in 75% of the patients (Krämer, Hillebrecht, Wang, et al. 2024b). Other features included microstomia, angular cheilitis, and pitted amelogenesis imperfecta (Krämer et al. 2020; Krämer, Hillebrecht, Wang, et al. 2024b). Blanchet et al. reported the cases of two brothers with KEB who underwent orthodontic treatment; one of the brothers exhibited a short U‐shaped dental arch with severe crowding and impacted upper canines (Blanchet et al. 2021). Given that only one of the two KEB participants was dentate, clinical findings are reported descriptively for this individual only: normal overjet and overbite, bilateral class III molar relationship, bilateral posterior crossbite, and coincident midlines, with pitting enamel abnormalities. Extraoral features were normal and symmetrical. Due to the insufficient sample size, KEB was excluded from inferential testing.

### 4.5 Clinical Implications

Orthodontics is listed as part of the common referral pathway for JEB and RDEB in the “Oral health care pathways for patients with EB: A position statement from the European reference network for rare skin diseases” (Krämer, Hillebrecht, Bekes, et al. 2024a). Despite this, orthodontic treatment for special care patients, and even more so for those with rare diseases, remains challenging due to several barriers, including the low prevalence of the condition, phenotypic variability, limited professional experience, and the economic burden (Arriagada‐Vargas et al. 2022; Véliz Méndez, Rotman, et al. 2022; Antonarakis and Kiliaridis 2021). Several case reports have demonstrated that orthodontic treatment is feasible in patients living with severe EB types, such as RDEB (Goldschmied 1999; Pacheco and Marques de Sousa Araugio 2008; Portillo Nava et al. 2014; Véliz et al. 2020; Véliz Méndez, Baeza, et al. 2022a, Véliz Méndez et al. 2023b), JEB (Véliz et al. 2024), and KEB (Blanchet et al. 2021). Additionally, the “Orthodontic Treatment in Patients with Epidermolysis Bullosa: Clinical Practice Guidelines” were published in 2025, seeking to decrease the barriers that people living with EB face when they seek specialist dental care (Véliz et al. 2025).

The findings of this study have direct clinical implications for screening and referral prioritization. The high prevalence of posterior anomalies in RDEB (74.5%, 95% CI 60.5–84.7; Cramér's *V* = 0.504) supports prioritizing transverse assessment during early dental evaluations in this subtype, with potential early interventions to prevent further skeletal restriction. The co‐occurrence of three or more dentofacial features in 53.1% of RDEB participants further underscores the complexity of this phenotype and the need for comprehensive orthodontic assessment. Further research is still needed, including to study the relationship between dentofacial anomalies and anatomical/functional anomalies in EB such as strictures, microstomia, and ankyloglossia.

### 4.6 Limitations

The present study has several limitations that should be considered when interpreting the results. Due to the complexity of oral conditions, particularly in RDEB, the use of dental impressions or intraoral scanners was not considered routinely feasible across the cohort, limiting crowding assessment to present/absent and preventing systematic posterior vertical assessment in patients with severe microstomia. Lateral cephalometric radiographs were only obtained when clinically indicated, precluding a systematic skeletal assessment across the full sample. Missing data in RDEB were predominantly attributable to prior tooth extraction, and should be considered potentially informative rather than random, as they reflect the severity of the disease in this subtype. Since EB is a rare disease with low prevalence and phenotypic variation, and despite the number of participants being remarkable for a rare disease (more than 50% of the universe); there are subtypes with few participants, making the information for those subtypes not fully representative of their characteristics. This is reflected in the statistical analysis, not considering more complex analyses due to statistical instability; but also leading us to use dichotomous analyses, which can simplify more complex phenomena. Although the reporting of confidence intervals and effect sizes partially addressed this limitation by providing estimates of uncertainty and clinical relevance, results for smaller subtypes should still be interpreted with caution. The absence of a healthy control group is inherent to the descriptive cross‐sectional design. Given the wide age range and the phenotypic heterogeneity of EB, a matched control group would be methodologically challenging to define meaningfully. Additionally, dividing the sample by age or gender would have increased the data dispersion. Some participants are very young, and therefore, certain characteristics, particularly skeletal ones, may not have fully manifested yet, as they are likely to appear after the completion of the dental eruption and the skeletal growth phase. Selection bias cannot be excluded, as all participants were recruited from one reference center. Future studies should consider multicentre analyses to limit this type of bias.

## 5 Conclusion

People living with EB present a high prevalence of dentofacial anomalies, varying with the type and subtype. Patients with RDEB showed the highest burden, with a significantly high prevalence of posterior transverse anomalies (74.5%, 95% CI 60.5–84.7; Cramér's *V* = 0.504) and increased overbite (Kruskal–Wallis test *χ* ^2^[3] = 9.62, *p* = 0.022, *ε* ^2^ = 0.106), with more than half of RDEB participants presenting three or more features of a coherent dentofacial phenotype. All JEB participants presented dental anomalies (100%, 95% CI 61.0–100.0, Cramér's *V* = 0.762), reinforcing the need for early and systematic dental screening in this subtype. Despite its limitations, this study provides the most comprehensive characterization to date of dentofacial anomalies across all EB types in a single cohort, offering valuable insights into an understudied aspect of this rare disease. Future studies should evaluate genotype–phenotype correlations, consider multicentre designs to reduce selection bias, and explore the relationship between dentofacial anomalies and orofacial functional impairment. Where clinically feasible, complementary assessment with intraoral scanners and cephalometric radiographs would enrich characterization of this group.

## Author Contributions

**Sebastián Véliz:** conceptualization, methodology, validation, formal analysis, investigation, data curation, writing – original draft, writing – review and editing, visualization. **Susanne Krämer:** conceptualization, methodology, validation, formal analysis, data curation, writing – original draft, writing – review and editing, visualization. **Colomba Besa‐Witto:** validation, formal analysis, investigation, data curation, writing – review and editing. **Pedro Diz‐Dios:** methodology, formal analysis, writing – review and editing, supervision.

## Funding

The authors have nothing to report.

## Ethics Statement

Ethical approval was obtained at the Ethics Committee of the North Metropolitan Health Service, Chile (002/2023).

## Consent

All participants were asked to sign a consent to participate. In the case of minors, both, the patient and their guardian, were asked to sign a consent to participate. The patients in Figure 1 also consented to include their photos and information in this article.

## Conflicts of Interest

The authors declare no conflicts of interest.

## Supporting information


Supporting File

## Data Availability

The data that support the findings of this study are available on request from the corresponding author. The data are not publicly available due to privacy or ethical restrictions.
