# Supplementary material for: Dentofacial Characteristics in Patients With Epidermolysis Bullosa—A Cross‐Sectional Study
Source: Clin Exp Dent Res. 2026 Oct 6;12(5):e70478. doi: 10.1002/cre2.70478 (PMC13640968; doi:10.1002/cre2.70478)
Supplement: Supplementary file 1 [file CRE2-12-e70478-s001.docx]

**SUPPLEMENTARY TABLES AND FIGURES**

## **Supplementary Table S1:** Fisher's exact test, Cramér's V and FDR-adjusted p-values for categorical variables

| **Variable** | **p (Fisher)** | **p (FDR)** | **Cramér's V** |
| --- | --- | --- | --- |
| Overjet | 0.712 | 0.712 | 0.133 |
| Molar relationship | 0.699 | 0.712 | 0.155 |
| Midline coincidence | 0.301 | 0.501 | 0.199 |
| Posterior transverse | <0.001 | <0.001 | 0.504 |
| Overbite | 0.016 | 0.052 | 0.320 |
| Crowding | 0.273 | 0.501 | 0.201 |
| Dental anomalies | <0.001 | <0.001 | 0.762 |
| Facial profile | 0.142 | 0.356 | 0.234 |
| Facial type | 0.570 | 0.712 | 0.143 |
| Facial symmetry | 0.466 | 0.666 | 0.163 |

KEB (n=2) excluded from inferential testing. p (FDR): Benjamini-Hochberg false discovery rate correction applied across all categorical outcomes. Cramér's V: 0.1 small, 0.3 medium, 0.5 large. EB: Epidermolysis Bullosa; EBS: EB Simplex; JEB: Junctional EB; DDEB: Dominant Dystrophic EB; RDEB: Recessive Dystrophic EB.

**Supplementary table S2:** Sagittal plane analysis

| SAGITTAL PLANE | | | | | | | | | | | | | | | | |
| --- | --- | --- | --- | --- | --- | --- | --- | --- | --- | --- | --- | --- | --- | --- | --- | --- |
| EB CLASSIFICATION | | | OVERJET | | | | | | | MOLAR RELATION | | | | | | |
| EB TYPE | EB SUBTYPE | N | V | M | Discrepancy in mm [Mean ± SD] | 1-4 mm [N (%)] | > 4 mm [N (%)] | 0 mm [N (%)] | < 0 mm [N (%)] | V | M | SIDE | CLASS I [N (%)] | CLASS II [N (%)] | CLASS III [N (%)] | ABSENT [N (%)] |
| EBS | LOCALISED | *3* | 3 | 0 | 3.3±1.5 | 2(66.7) | 1(33.3) | 0(0) | 0(0) | 3 | 0 | L | 1(33.3) | 1(33.3) | 1(33.3) | 0(0) |
|  |  |  |  |  |  |  |  |  |  | 3 | 0 | R | 2(66.7) | 0(0) | 1(33.3) | 0(0) |
|  | INTERMEDIATE | *18* | 17 | 1 | 2.4±1.9 | 13(76.5) | 1(5.9) | 2(11.8) | 1(5.9) | 17 | 1 | L | 5(29.4) | 1 (5.9) | 5(29.4) | 6(35.3) |
|  |  |  |  |  |  |  |  |  |  | 17 | 0 | R | 6(35.3) | 2(11.8) | 4(23.5) | 5(29.4) |
|  | IWMD | *5* | 5 | 0 | 2.4±1.9 | 4(80.0) | 0(0) | 0(0) | 1(20.0) | 5 | 0 | L | 1 (20.0) | 2 (40.0) | 2 (40.0) | 0(0) |
|  |  |  |  |  |  |  |  |  |  | 5 | 0 | R | 1 (20.0) | 2 (40.0) | 1 (20.0) | 1(20.0) |
|  | TOTAL EBS | *26* | 25 | 1 | 2.5±1.8 | 19(76.0) | 2(8.0) | 2(8.0) | 2(8.0) | 25 | 1 | L | 7 (28.0) | 4 (16.0) | 8 (32.0) | 6(24.0) |
|  |  |  |  |  |  |  |  |  |  | 25 | 1 | R | 9 (36.0) | 4 (16.0) | 6 (24.0) | 6(24.0) |
| JEB | INTERMEDIATE | *3* | 3 | 0 | 3.3±0.5 | 3(100) | 0(0) | 0(0) | 0(0) | 3 | 0 | L | 1 (33.3) | 1 (33.3) | 1 (33.3) | 0(0) |
|  |  |  |  |  |  |  |  |  |  | 3 | 0 | R | 1 (33.3) | 0(0) | 1 (33.3) | 1(33.3) |
|  | SEVERE | *3* | 3 | 0 | 3.6±1.5 | 2(66.6) | 1(33.3) | 0(0) | 0(0) | 3 | 0 | L | 1 (33.3) | 1 (33.3) | 1 (33.3) | 0(0) |
|  |  |  |  |  |  |  |  |  |  | 3 | 0 | R | 1 (33.3) | 1 (33.3) | 1 (33.3) | 0(0) |
|  | TOTAL JEB | *6* | 6 | 0 | 3.5±1.0 | 5(83.3) | 1(16.6) | 0(0) | 0(0) | 6 | 0 | L | 2 (33.3) | 2 (33.3) | 2 (33.3) | 0(0) |
|  |  |  |  |  |  |  |  |  |  | 6 | 0 | R | 2 (33.3) | 1 (16.7) | 2 (33.3) | 1(16.7) |
| DDEB | LOCALISED | *14* | 14 | 0 | 4±3.3 | 8 (57.1) | 4 (28.6) | 2 (14.3) | 0 (0) | 14 | 0 | L | 4 (28.6) | 5 (35.7) | 4 (28.6) | 1(7.1) |
|  |  |  |  |  |  |  |  |  |  | 14 | 0 | R | 5 (35.7) | 5 (35.7) | 4 (28.6) | 0(0) |
|  | PRURIGINOSA | *4* | 4 | 0 | 2.7±1.8 | 3 (75.0) | 0 (0) | 1 (25.0) | 0 (0) | 4 | 0 | L | 2 (50.0) | 0(0) | 1 (25.0) | 1 (25.0) |
|  |  |  |  |  |  |  |  |  |  | 4 | 0 | R | 1 (25.0) | 1 (25.0) | 1 (25.0) | 1(25.0) |
|  | TOTAL DDEB | *18* | 18 | 0 | 3.7±3.1 | 11(61.1) | 4 (22.2) | 3 (16.7) | 0 (0) | 18 | 0 | L | 6(33.3) | 5(27.8) | 5(27.8) | 2(11.1) |
|  |  |  |  |  |  |  |  |  |  | 18 | 0 | R | 6(33.3) | 6(33.3) | 5(27.8) | 1(5.6) |
| RDEB | LOCALISED | *1* | 1 | 0 | 4 | 1 (100) | 0 (0) | 0 (0) | 0 (0) | 1 | 0 | L | 0(0) | 0(0) | 0(0) | 1(100) |
|  |  |  |  |  |  |  |  |  |  | 1 | 0 | R | 0(0) | 0(0) | 0(0) | 1(100) |
|  | INTERMEDIATE | *21* | 18 | 3 | 1.7±3.2 | 11(61.1) | 2(11.1) | 0 (0) | 5 (27.8) | 15 | 6 | L | 1(6.7) | 3 (20.0) | 4 (26.7) | 7(46.7) |
|  |  |  |  |  |  |  |  |  |  | 15 | 6 | R | 4(26.7) | 1(6.7) | 2(13.3) | 8(53.3) |
|  | SEVERE | *25* | 23 | 2 | 2.4±2.9 | 14(60.9) | 4(17.4) | 1 (4.3) | 4 (17.4) | 25 | 0 | L | 1 (4.0) | 5 (20.0) | 5 (20.0) | 14 (56.0) |
|  |  |  |  |  |  |  |  |  |  | 25 | 0 | R | 3 (12.0) | 4 (16.0) | 5 (20.0) | 13(52.0) |
|  | INVERSA | *2* | 1 | 1 | 3 | 1 (100) | 0 (0) | 0 (0) | 0 (0) | 2 | 0 | L | 0(0) | 0(0) | 0(0) | 2(100) |
|  |  |  |  |  |  |  |  |  |  | 2 | 0 | R | 1 (50.0) | 0(0) | 0(0) | 1(50.0) |
|  | TOTAL RDEB | *49* | 43 | 6 | 2.2±2.9 | 27(62.8) | 6 (14.0) | 1 (2.3) | 9 (20.9) | 43 | 6 | L | 2(4.7) | 8(18.6) | 9(20.9) | 24(55.8) |
|  |  |  |  |  |  |  |  |  |  | 43 | 6 | R | 8(18.6) | 5(11.6) | 7(16.3) | 23(53.5) |
| KEB | KINDLER | *2* | 1 | 1 | 1 | 1 (100) | 0 (0) | 0 (0) | 0 (0) | 2 | 0 | L | 0(0) | 0(0) | 1 (50.0) | 1(50.0) |
|  |  |  |  |  |  |  |  |  |  | 2 | 0 | R | 0(0) | 0(0) | 1 (50.0) | 1(50.0) |
|  | TOTAL | 101 | 93 | 8 | 2.6±2.6 | 63 (67.7) | 13 (14.0) | 6 (6.5) | 11 (11.8) | 94 | 7 | L | 17 (18.1) | 19 (20.2) | 25 (26.6) | 33(35.1) |
|  |  |  |  |  |  |  |  |  |  | 94 | 7 | R | 25(26.6) | 16(17.0) | 21(22.3) | 32(34.0) |
|  |  |  |  |  |  |  |  |  |  | 188 | 14 | TOTAL | 42(22.3) | 35(18.6) | 46(24.4) | 65(34.6) |

TABLE 1: EB: Epidermolysis Bullosa; EBS: Epidermolysis Bullosa Simplex; JEB: Junctional EB; DDEB: Dominant Dystrophic EB; RDEB; Recessive Dystrophic EB; KEB: Kindler EB; IWMD: Intermediate With Muscular Dystrophy EB; L: Left side; R: Right side. V: Valid data. M: Missing data; SD: Standard deviation.

## **Supplementary Table S3:** Independent-Samples Kruskal-Wallis Test – Overjet by EB type

| **Test summary** | **Value** |
| --- | --- |
| Total N | 93 |
| Test Statistic | 3.559 |
| Degrees of Freedom | 3 |
| Asymptotic Sig. (2-sided) | 0.313 |
| Epsilon-squared (ε²) | 0.039 |

| **Pairwise  post-hoc comparisons** | **Adj. Sig. (Bonferroni)** |
| --- | --- |
| EBS – JEB | 1.000 |
| EBS – DDEB | 1.000 |
| EBS – RDEB | 1.000 |
| JEB – DDEB | 1.000 |
| JEB – RDEB | 1.000 |
| DDEB – RDEB | 0.888 |

All EB types included (KEB n=2 included for completeness). Test statistic adjusted for ties. ε²: 0.01 small, 0.06 medium, 0.14 large. EB: Epidermolysis Bullosa; EBS: EB Simplex; JEB: Junctional EB; DDEB: Dominant Dystrophic EB; RDEB: Recessive Dystrophic EB; KEB: Kindler EB.

**Supplementary table S4:** Transversal plane analysis.

|  |  |  | TRANSVERSAL PLANE | | | | | | | | | | |
| --- | --- | --- | --- | --- | --- | --- | --- | --- | --- | --- | --- | --- | --- |
| EB CLASSIFICATION | | | MIDLINE COINCIDENCE | | | | POSTERIOR TRANSVERSE RELATIONSHIP | | | | | | |
| EB TYPE | EB SUBTYPE | N | V | M | Coinciding [N (%)] | Non-coinciding [N (%)] | V | M | Absence of malocclusion  [N (%)] | Unilateral crossbite  [N (%)] | Bilateral crossbite  [N (%)] | Unilateral scissors [N (%)] | Bilateral scissors [N (%)] |
| EBS | LOCALISED | 3 | 3 | 0 | 2(66.7) | 1(33.3) | 3 | 0 | 3(100) | 0(0) | 0(0) | 0(0) | 0(0) |
|  | INTERMEDIATE | 18 | 17 | 1 | 13(76.5) | 4(23.5) | 18 | 0 | 13(72.2) | 2(11.1) | 3(16.7) | 0(0) | 0(0) |
|  | IWMD | 5 | 4 | 1 | 2(50.0) | 2(50.0) | 5 | 0 | 5(100) | 0(0) | 0(0) | 0(0) | 0(0) |
|  | TOTAL EBS | 26 | 24 | 2 | 17(70.8) | 7(29.2) | 26 | 0 | 21(80.8) | 2(7.7) | 3(11.5) | 0(0) | 0(0) |
| JEB | INTERMEDIATE | 3 | 3 | 0 | 1(33.3) | 2(66.7) | 3 | 0 | 2(66.7) | 0(0) | 1(33.3) | 0(0) | 0(0) |
|  | SEVERE | 3 | 3 | 0 | 3(100) | 0(0) | 3 | 0 | 3(100) | 0(0) | 0(0) | 0(0) | 0(0) |
|  | TOTAL JEB | 6 | 6 | 0 | 4(66.7) | 2(33.3) | 6 | 0 | 5(83.3) | 0(0) | 1(16.7) | 0(0) | 0(0) |
| DDEB | LOCALISED | 14 | 14 | 0 | 9(64.3) | 5(35.7) | 14 | 0 | 9(64.3) | 3(21.4) | 2(14.3) | 0(0) | 0(0) |
|  | PRURIGINOSA | 4 | 4 | 0 | 1(25.0) | 3(75.0) | 4 | 0 | 2(50.0) | 1(25.0) | 0(0) | 1(25.0) | 0(0) |
|  | TOTAL DDEB | 18 | 18 | 0 | 10(55.6) | 8(44.4) | 18 | 0 | 11(61.1) | 4(22.2) | 2(11.1) | 1(5.6) | 0(0) |
| RDEB | LOCALISED | 1 | 1 | 0 | 0(0) | 1(100) | 1 | 0 | 1(100) | 0(0) | 0(0) | 0(0) | 0(0) |
|  | INTERMEDIATE | 21 | 19 | 2 | 10(52.6) | 9(47.4) | 20 | 1 | 6(30.0) | 7(35.0) | 6(30.0) | 0(0) | 1(5.0) |
|  | SEVERE | 25 | 23 | 2 | 11(47.8) | 12(52.2) | 25 | 0 | 5(20.0) | 10(40.0) | 9(36.0) | 0(0) | 1(4.0) |
|  | INVERSA | 2 | 1 | 1 | 0(0) | 1(100) | 1 | 1 | 0(0) | 1(100) | 0(0) | 0(0) | 0(0) |
|  | TOTAL RDEB | 49 | 44 | 5 | 21(47.7) | 23(52.3) | 47 | 2 | 12(25.5) | 18(38.3) | 15(31.9) | 0(0) | 2(4.3) |
| KEB | KINDLER | 2 | 1 | 1 | 1(100) | 0(0) | 1 | 1 | 0(0) | 0(0) | 1(100) | 0(0) | 0(0) |
|  | TOTAL | 101 | 93 | 8 | 53(57.0) | 40(43.0) | 98 | 3 | 49(50.0) | 24(24.5) | 22(22.4) | 1(1.0) | 2(2.0) |

EB: Epidermolysis Bullosa; EBS: Epidermolysis Bullosa Simplex; JEB: Junctional EB; DDEB: Dominant Dystrophic EB; RDEB; Recessive Dystrophic EB; KEB: Kindler EB; IWMD: Intermediate with Muscular Dystrophy EB; V: Valid data. M: Missing data.

**Supplementary table S5:** Vertical plane and intramaxillary analysis.

|  |  |  | VERTICAL PLANE | | | | | | | INTRAMAXILLARY ANOMALIES | | | | | | | | |
| --- | --- | --- | --- | --- | --- | --- | --- | --- | --- | --- | --- | --- | --- | --- | --- | --- | --- | --- |
| EB CLASSIFICATION | | | OVERBITE | | | | | | | CROWDING | | DENTAL ANOMALIES | | | | | | |
| EB TYPE | EB SUBTYPE | N | V | M | Discrepancy in mm [Mean ± SD] | 1-4 mm [N (%)] | Open bite [N (%)] | Edge-to-edge [N (%)] | Deep bite [N (%)] | V | Crowding [N (%)] | V | M | None [N (%)] | Agenesis [N (%)] | Eruption complication [N (%)] | Supernumerary [N (%)] | Shape [N (%)] |
| EBS | LOCALISED | 3 | 3 | 0 | 2.3±1.5 | 3(100) | 0(0) | 0(0) | 0(0) | 3 | 1(33.3) | 3 | 0 | 3(100) | 0(0) | 0(0) | 0(0) | 0(0) |
|  | INTERMEDIATE | 18 | 17 | 1 | 2.0±1.2 | 15(88.2) | 0(0) | 2(11.8) | 0(0) | 18 | 11(61.1) | 18 | 0 | 17(94.4) | 0(0) | 0(0) | 1(5.6) | 0(0) |
|  | IWMD | 5 | 5 | 0 | 3.4±3.7 | 4(80.0) | 0(0) | 0(0) | 1(20.0) | 5 | 2(40.0) | 5 | 0 | 4(80.0) | 1(20.0) | 0(0) | 0(0) | 0(0) |
|  | TOTAL EBS | 26 | 25 | 1 | 2.3±1.9 | 22(88.0) | 0(0) | 2(8.0) | 1(4.0) | 26 | 14(53.8) | 26 | 0 | 24(92.3) | 1(3.8) | 0(0) | 1(3.8) | 0(0) |
| JEB | INTERMEDIATE | 3 | 3 | 0 | 1.6±4.5 | 1(33.3) | 1(33.3) | 0(0) | 1(33.3) | 3 | 1(33.3) | 3 | 0 | 0(0) | 1(33.3) | 3(100) | 0(0) | 3(100) |
|  | SEVERE | 3 | 3 | 0 | 2.3±0.5 | 3(100) | 0(0) | 0(0) | 0(0) | 3 | 1(33.3) | 3 | 0 | 0(0) | 0(0) | 3(100) | 0(0) | 3(100) |
|  | TOTAL JEB | 6 | 6 | 0 | 2.0±2.8 | 4(66.7) | 1(16.7) | 0(0) | 1(16.7) | 6 | 2(33.3) | 6 | 0 | 0(0) | 1(16.7) | 6(100) | 0(0) | 6(100) |
| DDEB | LOCALISED | 14 | 14 | 0 | 2.5±2.7 | 9(64.3) | 1(7.1) | 2(14.3) | 2(14.3) | 14 | 6(42.9%) | 14 | 0 | 14(100) | 0(0) | 0(0) | 0(0) | 0(0) |
|  | PRURIGINOSA | 4 | 4 | 0 | 2.5±2.0 | 2(50.0) | 0(0) | 1(25.0) | 1(25.0) | 4 | 2(50.0) | 4 | 0 | 3(75.0) | 0(0) | 1(25.0) | 0(0) | 0(0) |
|  | TOTAL DDEB | 18 | 18 | 0 | 2.5±2.5 | 11(61.1) | 1(5.6) | 3(16.7) | 3(16.7) | 18 | 8(44.4) | 18 | 0 | 17(94.4) | 0(0) | 1(5.6) | 0(0) | 0(0) |
| RDEB | LOCALISED | 1 | 1 | 0 | 3.0 | 1(100) | 0(0) | 0(0) | 0(0) | 1 | 0(0) | 1 | 0 | 1(100) | 0(0) | 0(0) | 0(0) | 0(0) |
|  | INTERMEDIATE | 21 | 18 | 3 | 2.7±2.1 | 14(77.8) | 0(0) | 2(11.9 | 2(11.9 | 21 | 14(66.7) | 20 | 1 | 20(100) | 0(0) | 0(0) | 0(0) | 0(0) |
|  | SEVERE | 25 | 23 | 2 | 5.0±2.9 | 7(30.4) | 0(0) | 1(4.3) | 15(65.2) | 25 | 18(72.0) | 25 | 0 | 24(96.0) | 0(0) | 1(4.0) | 0(0) | 0(0) |
|  | INVERSA | 2 | 1 | 1 | 6.0 | 0(0) | 0(0) | 0(0) | 1(100) | 2 | 0(0) | 2 | 0 | 2(100) | 0(0) | 0(0) | 0(0) | 0(0) |
|  | TOTAL RDEB | 49 | 43 | 6 | 4.0±2.7 | 22(51.2) | 0(0) | 3(7.0) | 18(41.9) | 49 | 32(65.3) | 48 | 1 | 47(97.9) | 0(0) | 1(2.1) | 0(0) | 0(0) |
| KEB | KINDLER | 2 | 1 | 1 | 1.0 | 1(100) | 0(0) | 0(0) | 0(0) | 2 | 0(0) | 1 | 1 | 1(100) | 0(0) | 0(0) | 0(0) | 0(0) |
|  | TOTAL | 101 | 93 | 8 | 3.1±2.6 | 60(64.5) | 2(2.2) | 8(8.6) | 23(24.7) | 101 | 56(55.4) | 99 | 2 | 89 (89.9) | 2(2.0) | 8(8.0) | 1(1.0) | 6(6.0) |

EB: Epidermolysis Bullosa; EBS: Epidermolysis Bullosa Simplex; JEB: Junctional EB; DDEB: Dominant Dystrophic EB; RDEB; Recessive Dystrophic EB; KEB: Kindler EB; IWMD: Intermediate with Muscular Dystrophy EB; V: Valid data. M: Missing data; SD: Standard deviation.

## **Supplementary Table S6:** Independent-Samples Kruskal-Wallis Test – Overbite by EB type

| **Test summary** | **Value** |
| --- | --- |
| Total N | 92 |
| Test Statistic | 9.621 |
| Degrees of Freedom | 3 |
| Asymptotic Sig. (2-sided) | 0.022 |
| Epsilon-squared (ε²) | 0.106 |

(Bonferroni)

| **Pairwise  post-hoc comparisons** | **Adj. Sig. (Bonferroni)** |
| --- | --- |
| EBS – JEB | 1.000 |
| EBS – DDEB | 1.000 |
| EBS – RDEB | 0.025 |
| JEB – DDEB | 1.000 |
| JEB – RDEB | 0.877 |
| DDEB – RDEB | 0.585 |

KEB (n=2) excluded from inferential testing. Test statistic adjusted for ties. ε²: 0.01 small, 0.06 medium, 0.14 large. EB: Epidermolysis Bullosa; EBS: EB Simplex; JEB: Junctional EB; DDEB: Dominant Dystrophic EB; RDEB: Recessive Dystrophic EB.

**Supplementary table S7:** Extraoral assessment.

|  |  |  | EXTRAORAL ASSESSMENT | | | | | | | |
| --- | --- | --- | --- | --- | --- | --- | --- | --- | --- | --- |
| EB CLASSIFICATION | | | FACIAL PROFILE | | | FACIAL TYPE | | | FRONTAL SYMMETRY | |
| EB TYPE | EB SUBTYPE | N | Straight [N (%)] | Concave [N (%)] | Convex [N (%)] | Normodivergent [N (%)] | Hypodivergent [N (%)] | Hyperdivergent [N (%)] | Symmetrical [N (%)] | Asymmetrical [N (%)] |
| EBS | LOCALISED | 3 | 3(100) | 0(0) | 0(0) | 3(100) | 0(0) | 0(0) | 3(100) | 0(0) |
|  | INTERMEDIATE | 18 | 7(38.9) | 9(50) | 2(11.1) | 4(22.2) | 9(50.0) | 5(27.8) | 18(100) | 0(0) |
|  | IWMD | 5 | 1(20.0) | 3(60.0) | 1(20.0) | 2(40.0) | 0(0) | 3(60.0) | 2(40.0) | 3(60.0) |
|  | TOTAL EBS | 26 | 11(42.3) | 12(46.2) | 3(11.5) | 9(34.6) | 9(34.6) | 8(30.8) | 23(88.5) | 3(11.5) |
| JEB | INTERMEDIATE | 3 | 2(66.6) | 0(0) | 1(33.3) | 2(66.7) | 0(0) | 1(33.3) | 2(66.7) | 1(33.3) |
|  | SEVERE | 3 | 2(66.6) | 1(33.3) | 0(0) | 0(0) | 2(66.7) | 1(33.3) | 3(100) | 0(0) |
|  | TOTAL JEB | 6 | 4(66.6) | 1(16.6) | 1(16.6) | 2(33.3) | 2(33.3) | 2(33.3) | 5(83.3) | 1(16.7) |
| DDEB | LOCALISED | 14 | 6(42.9) | 4(28.6) | 4(28.6) | 2(14.3) | 5(35.7) | 7(50.0) | 13(92.9) | 1(7.1) |
|  | PRURIGINOSA | 4 | 4(100) | 0(0) | 0(0) | 1(25.0) | 2(50.0) | 1(25.0) | 4(100) | 0(0) |
|  | TOTAL DDEB | 18 | 10(55.6) | 4(22.2) | 4(22.2) | 3(16.7) | 7(38.9) | 8(44.4) | 17(94.4) | 1(5.6) |
| RDEB | LOCALISED | 1 | 0(0) | 0(0) | 1(100) | 0(0) | 0(0) | 1(100) | 0(0) | 1(100) |
|  | INTERMEDIATE | 21 | 6(28.6) | 12(57.1) | 3(14.3) | 5(23.8) | 11(52.4) | 5(23.8) | 16(76.2) | 5(23.8) |
|  | SEVERE | 25 | 9(36.0) | 8(32.0) | 8(32.0) | 7(28.0) | 15(60.0) | 3(12.0) | 21(84.0) | 4(16.0) |
|  | INVERSA | 2 | 0(0) | 1(50.0) | 1(50.0) | 0(0) | 1(50.0) | 1(50.0) | 2(100) | 0(0) |
|  | TOTAL RDEB | 49 | 15 (30.6) | 21(42.9) | 13(26.5) | 12(24.5) | 27(55.1) | 10(20.4) | 39(79.6) | 10(20.4) |
| KEB | KINDLER | 2 | 1(50.0) | 1(50.0) | 0(0) | 2(100) | 0 (0) | 0 (0) | 2(100) | 0(0) |
|  | TOTAL | 101 | 41(40.6) | 39(38.6) | 21(20.8) | 28(27.7) | 45(44.6) | 28(27.7) | 86(85.1) | 15(14.9) |

EB: Epidermolysis Bullosa; EBS: Epidermolysis Bullosa Simplex; JEB: Junctional EB; DDEB: Dominant Dystrophic EB; RDEB; Recessive Dystrophic EB; KEB: Kindler EB; IWMD: Intermediate with Muscular Dystrophy EB. V: Valid data. M: Missing data.

**Supplementary Table S8.** Sensitivity analysis: full sample vs participants aged ≥12 years

Categorical variables

| **Variable** | **n (full)** | **Cramér's V (full)** | **p (FDR), full** | **n (≥12y)** | **Cramér's V (≥12y)** | **p (FDR), ≥12y** |
| --- | --- | --- | --- | --- | --- | --- |
| Overjet | 92 | 0.133 | 0.712 | 70 | 0.201 | 0.527 |
| Molar relationship | 66 | 0.155 | 0.712 | 52 | 0.168 | 0.876 |
| Midline coincidence | 92 | 0.199 | 0.501 | 69 | 0.335 | 0.124 |
| Posterior transverse | 97 | 0.504 | <0.001 | 73 | 0.402 | 0.023 |
| Overbite (categorical) | 92 | 0.320 | 0.052 | 70 | 0.371 | 0.046 |
| Crowding | 99 | 0.201 | 0.501 | 75 | 0.156 | 0.805 |
| Dental anomalies | 98 | 0.762 | <0.001 | 75 | 0.695 | 0.003 |
| Facial profile | 99 | 0.234 | 0.356 | 75 | 0.220 | 0.440 |
| Facial type | 99 | 0.143 | 0.712 | 75 | 0.145 | 0.876 |
| Facial symmetry | 99 | 0.163 | 0.666 | 75 | 0.250 | 0.440 |

Continuous variable

| **Variable** | **n (full)** | **χ² (full)** | **p (full)** | **ε² (full)** | **n (≥12y)** | **χ² (≥12y)** | **p (≥12y)** | **ε² (≥12y)** |
| --- | --- | --- | --- | --- | --- | --- | --- | --- |
| Overjet (continuous, mm) | 92 | 3.559 | 0.313 | 0.039 | 70 | 4.481 | 0.214 | 0.065 |
| Overbite (continuous, mm) | 92 | 9.621 | 0.022 | 0.106 | 70 | 11.565 | 0.009 | 0.168 |

Analyses restricted to participants aged ≥12 years (established permanent dentition), excluding KEB. Cramér's V: 0.1 small, 0.3 medium, 0.5 large. ε²: 0.01 small, 0.06 medium, 0.14 large. p (FDR): Benjamini-Hochberg-adjusted across 10 categorical outcomes shown; p-values for continuous variables (Kruskal-Wallis) are unadjusted.

**Supplementary figure S1:** Flow diagram of participants.

EB: Epidermolysis Bullosa; EBS: Epidermolysis Bullosa Simplex; JEB: Junctional EB; DDEB: Dominant Dystrophic EB; RDEB; Recessive Dystrophic EB; KEB: Kindler EB..

**Supplementary figure S2:** Co-occurrence of proposed RDEB phenotype features.

RDEB; Recessive Dystrophic EB. Proposed RDEB phenotype: Posterior transverse anomaly + Altered overbite + Altered profile + Altered facial type.
